# Polymer Composites with Self-Regulating Temperature Behavior: Properties and Characterization

**DOI:** 10.3390/ma16010157

**Published:** 2022-12-24

**Authors:** Radu Setnescu, Eduard-Marius Lungulescu, Virgil Emanuel Marinescu

**Affiliations:** 1 National Institute for Research and Development in Electrical Engineering ICPE-CA, 313 Splaiul Unirii, 030138 Bucharest, Romania; 2 Department of Advanced Technologies, Faculty of Sciences and Arts, Valahia University of Târgoviște, 13 Aleea Sinaia, 130004 Târgoviște, Romania

**Keywords:** polymeric composites, PTC, NTC, self-regulating temperatures, conductive polymers

## Abstract

A novel conductive composite material with homogeneous binary polymer matrix of HDPE (HD) and LLDPE (LLD), mixed with conductive filler consisting of carbon black (CB) and graphite (Gr), was tested against a HDPE composite with a similar conductive filler. Even the concentration of the conductive filler was deliberately lower for (CB + Gr)/(LLD + HD), and the properties of this composite are comparable or better to those of (CB + Gr)/HD. The kinetic parameters of the ρ-T curves and from the DSC curves indicate that the resistivity peak is obtained when the polymer matrix is fully melted. When subjected to repeated thermal cycles, the composite (CB + Gr)/(LLD + HD) presented a better electrical behavior than composite CB + Gr)/HD, with an increase in resistivity (ρ_max_) values with the number of cycles, as well as less intense NTC (*Negative Temperature Coefficient*) effects, both for the crosslinked and thermoplastic samples. Radiation crosslinking led to increased ρ_max_ values, as well as to inhibition of NTC effects in both cases, thus having a clear beneficial effect. Limitation effects of surface temperature and current intensity through the sample were observed at different voltages, enabling the use of these materials as self-regulating heating elements at various temperatures below the melting temperature. The procedure based on physical mixing of the components appears more efficient in imparting lower resistivity in solid state and high PTC (*Positive Temperature Coefficient*) effects to the composites. This effect is probably due to the concentration of the conductive particles at the surface of the polymer domains, which would facilitate the formation of the conductive paths. Further work is still necessary to optimize both the procedure of composite preparation and the properties of such materials.

## 1. Introduction

Electrically conductive polymer composites (some remarkable pioneering work in this field should be mentioned, se for example [1,2,3]) attracted much attention due to their remarkable properties, which combine economic processing, good mechanical and chemical properties, and wide range of electrical properties, which can be finely controlled by adjusting the composition—polymer matrix and conductive filler [4,5]. Due to their properties, these materials exhibit high functionality and smartness in various technical applications comprising conductive coatings, electromagnetic shielding, electronic packaging flexible displays, sensors, etc. [6,7,8,9,10]. Various conductive powders or fiber materials/nanomaterials [7,11,12] can be used as conductive fillers, the carbon ones being of great interest due to low cost, good electrical properties, acceptable compatibility with polymer matrix, low density, and corrosion resistance. Carbon black [8,13,14], carbon fibers [15,16], graphite [17]), graphene [9,18], reduced graphene oxide [19], CNT [11,16,18], and other carbon materials were studied in order to obtain adequate properties for a wide range of applications. Among them, the composites exhibiting so-called *Positive Temperature Coefficient* (of resistivity) effect, abbreviated PTC [1] or PTCR [13], are of special interest for specific applications claiming self-limitation (of current) or switching (conductive/resistive) behaviors, such as self-regulating heating elements, current or voltage protections or temperature sensors [15,17]. Another effect occurring in conductive composites is known as NTC (*Negative Temperature Coefficient*), i.e., decrease in resistivity as the temperature increase. Note that these effects may occur in the same conductive composite, depending on several factors, such as temperature, polymer state, distribution of the filler, etc. Even there are some applications for the NTC materials, for example as temperature sensors, the PTC effect have a wider field of applications due to resistance increase with temperature rise, resulting in limitation of both the current and temperature of the device [6].

Due to its high crystallinity, HDPE is a good candidate for high PTC effects, but the mechanical rigidity of the resulting composites would be high at higher filler contents (15–25%), specific for self-regulating heating materials, are used. Blends of HDPE with other (miscible, low crystallinity) polymers, such as EVA (Ethylene-vinyl acetate), LDPE (Low-Density Polyethylene), would improve the mechanical properties of the composites [20]. In general, improvement of the properties of PTC materials requires compositional optimization of both polymer matrix and conductive fillers [17]. Polymer blends and synergic mixtures of different conductive powders are of actual interest in this direction [5,17,21,22].

The aim of this work was to study the PTC properties of a composite with a matrix consisting of a miscible blend of two polymers, LLDPE and HDPE, in comparison to HDPE only. This type of blend was less studied in composites in general [23], but it would present some interesting properties for PTC composites. Even a decrease in PTC intensity due to blending with lower crystallinity LLDPE is expected if we consider the hypothesis that some amorphous non-crystalline regions of PE remain unperturbed in the vicinity of CB particles [24], and LLDPE would be of interest due to its regular structure. In addition, the studied composites were prepared using a procedure based on powder mixing in a dry state, which appears to present some advantages. The influence of the processing technology and matrix composition is discussed in comparison with a composite having similar conductive filler but HDPE matrix.

## 2. Materials and Methods

### 2.1. Materials

Blends of polymer and conductive particles were prepared by dry mixing of different amounts of polymer powders, antioxidants, and conductive carbon powders.

HDPE, type ELTEX A3180PN1852 from Ineos, Vienna, Austria, and LLDPE, type RX 806 Natural from Resinex, Bucharest, Romania were used, as received, for polymer matrices. The weight ratio LLDPE: HDPE was 0.6:1.

The list of abbreviations used in this manuscript are presented in Table 1.

As conductive fillers, carbon black (FEF type) and natural graphite (CR10) were used (see [22] for more details).

The composite with HDPE matrix was codified (CB + Gr)/HD, meaning that it contains an HDPE matrix and carbon black (CB) and graphite (Gr) as conductive fillers. Similarly, the composite named below (CB + Gr)/(LLD + HD) has a binary blend matrix consisting in LLDPE and HDPE and a mixture of carbon black and graphite as conductive filler.

The procedures to prepare the above-mentioned composites (CB + Gr)/HD and (CB + Gr)/(LLD + HD) are described in applications [21,25]. In essence, the composite (CB + Gr)/HD is prepared by dry mixing of the powder components followed by extruder mixing (in melts state) and molding. The composite (CB + Gr)/(LLD + HD) was prepared by intensive dry mixing of the components followed by molding. To prove the effectiveness of this second procedure, the total concentration of the conductive filler was lower in (CB + Gr)/(LLD + HD), namely, 18.6%, as compared to 24.2% in (CB + Gr)/HD, but the weight ratio CB/Gr was the same, namely 4.5:1. Similar counterparts of these composites were prepared by melt extrusion + molding for (CB + Gr)/(LLD + HD) and intensive dry mixing + molding for (CB + Gr)/HD compounds.

### 2.2. Instruments and Methods

The pellets resulted from extrusion mixing or the powder mixture was formed as plates of 120 × 100 × 0.8 mm using a conventional heated mold with controlled temperature and a laboratory hydraulic press. Basically, the following molding conditions were used: temperature 170 °C (heating rate by 5 °C/min), holding time at maximum temperature 2 min, chilling under pressure (2.5 °C/min).

For radiation crosslinking, the samples were wrapped in aluminum foil and exposed to γ-rays in Ob-Servo Sanguis laboratory irradiator (Institute of Isotopes, Budapest, Hungary) equipped with ^60^Co isotope (dose rate ~0.7 kGy/h, integral dose 150 kGy) in presence of air at room temperature.

SEM micrographs were recorded on FESEM scanning electron microscope dual beam type, model Auriga (Carl Zeiss SMT, Oberkochen, Germany). Additionally, a secondary electron detector Everhart-Thornley type within the chamber and In-Lens in column detector for ultra-topography images were employed. The magnification range was 1000x–50,000x with approximately 5 mm working distance. Various magnification SEM images (basically 1 kx, 5 kx and 20 kx) were taken out in different representative regions of each sample in order to better understanding their morphologies.

The volume (ρ_V_) and surface (ρ_S_) resistivities were measured at room temperature on 100 × 100 × 0.7 mm plates, using a Keithley electrometer, following a standard procedure.

**Figure 1:**
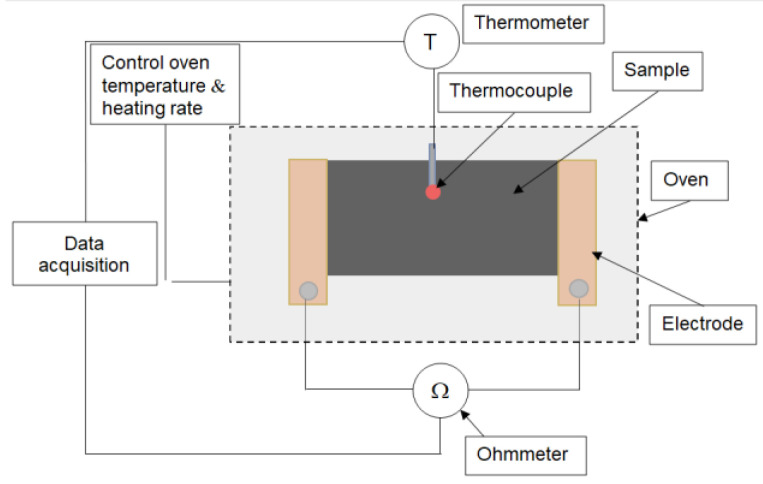
Schematic setup for ρ-T measurements.

The variation of the resistivity with temperature was measured on small samples (chips of 35 × 25 × 0.7 mm, with 25 mm distance between the flat electrodes, Figure 1) using a digital multimeter for electrical resistance and a thermocouple in contact with the sample surface.

The slope of ρ increase (the rate of resistivity increase on the heating curve) was calculated with the Formula (1):

(1) $$\mathrm{slope}=\frac{\rho_{2}-\rho_{1}}{\rho_{\max}}\cdot \frac{1}{T_{2}-T_{1}}$$

Similar formula was used to describe the resistivity decrease (on NTC regions) or to calculate the slopes on the cooling curves.

**Figure 2:**
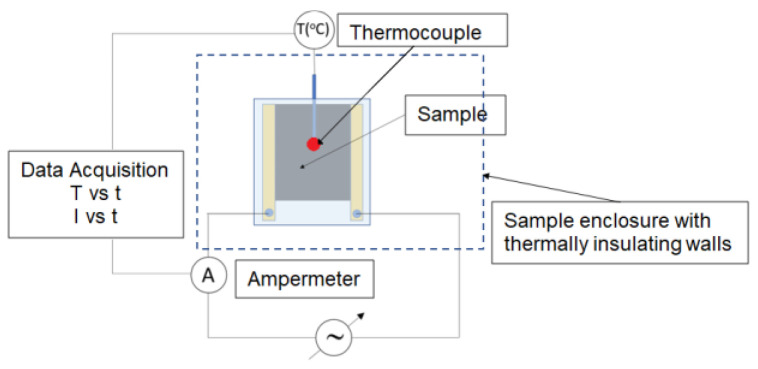
Schematic setup for T_s_ vs. time and I vs. time measurements.

A similar setup as above was used for measurements of the temperature on the sample surface (T_s_, Figure 2). A thermo-insulating enclosure was used instead of the oven. An amperemeter was integrated within the circuit enabled the measurement of the absorbed electrical power.

**Figure 3:**
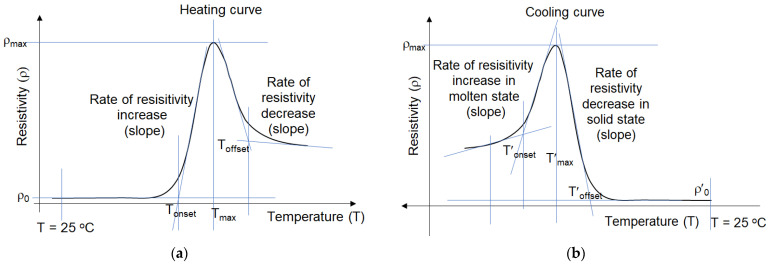
Typical ρ-T heating (**a**) and ρ-T cooling (**b**) curves and parameters used for kinetic characterization of resistivity vs. temperature.

A typical curve of ρ vs. T upon heating, with the parameters characterizing the kinetics of the process is shown in Figure 3. The cooling curve has similar shape, but is inversed, due to temperature decrease.

DSC measurements were performed in non-isothermal mode, under either air (oxidation tests) or nitrogen (melting/crystallinity tests), using a DSC 131 evo instrument (Setaram, Lyon, France). The parameters describing the oxidation (OOT, oxidation rate, oxidation heat) were calculated from the thermograms as described in references [26,27]. The melting/crystallinity peaks, the melting/ crystallization temperatures, and the thermal effects associated to either melting or crystallization were calculated as described earlier in the reference [22]. The crystalline content (*X_c_*) of the material was calculated by equations presented in reference [22], using a value of 279 J/g for melting enthalpy of totally crystalline polyethylene [28].

## 3. Results and Discussion

### 3.1. SEM Characterization

**Figure 4:**
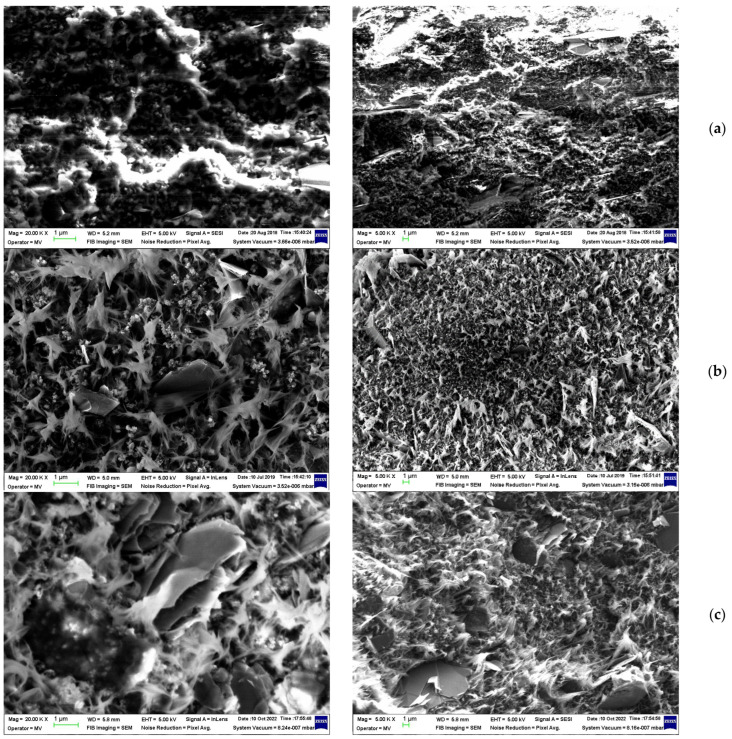
SEM images of (CB + Gr)/HD composite stored for different periods at r.t.: (**a**) freshly prepared sample; (**b**) stored for one year; (**c**) stored for four years. In the first column, the magnification is 20 kx while in the second one, it is 5 kx.

**Figure 5:**
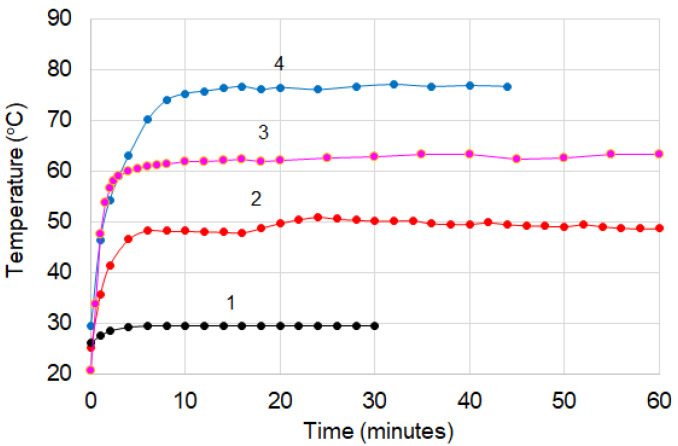
The surface temperature (T_s_) vs. time for (CB + Gr)/HD composite with different histories/treatments: 1—freshly prepared by melt extrusion, unirradiated; 2—prepared by melt extrusion and irradiated after 2 months after preparation; 3—prepared by melt extrusion, stored as pellets for 3 years, then formed by press molding and irradiated; and 4—freshly prepared by physical mixing of the components and press molding, unirradiated.

The Figure 4 presents the effect of long-term storage at room temperature (r.t.) on a (CB + Gr)/HD (24.2% conductive charge) sample prepared by melt extrusion followed by press molding (at 150 °C). It can be seen that the “luminosity”, due to charge accumulation, on the sample surface during the SEM measurement decreased clearly from the freshly prepared sample to those stored for one year or more. It can be said that the stored material is better structured in the sense that for the long-term stored materials, and the conductive particles are more segregated from the polymer matrix, enabling the formation of more conductive channels and, as a result, higher conductivity. Resistivity measurements confirmed this interpretation: while the freshly prepared sample presented rather low conductivity [29], the stored samples presented considerably higher conductivity. This is seen, for example, in the composite (CB + Gr)/HD, which presented a decrease from 6.5 × 10^9^ Ω⋅m for the freshly prepared sample [29] to 9.8 × 10^3^ Ω⋅m after four years of storage. The surface temperature (T_s_) of similar samples measured at different moments followed a similar trend (Figure 5): subjected to a same voltage, the stored sample presented significantly higher T_s_ values (on the plateau region) as compared to the freshly prepared one, indicating higher Joule effect due to increased current, which traverses the stored sample. The effects of irradiation and preparation procedure are also illustrated in Figure 4. The thermal effect of the studied composites is discussed in more detail in Section 3.3.

**Figure 6:**
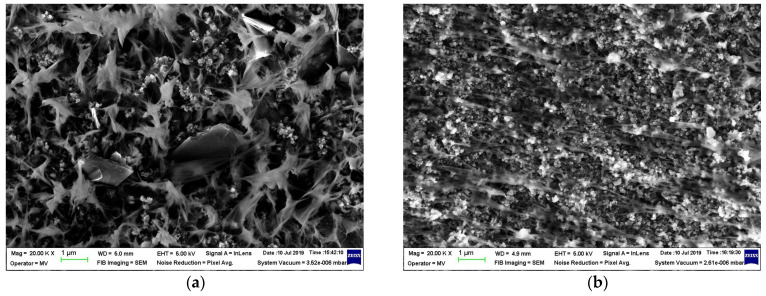

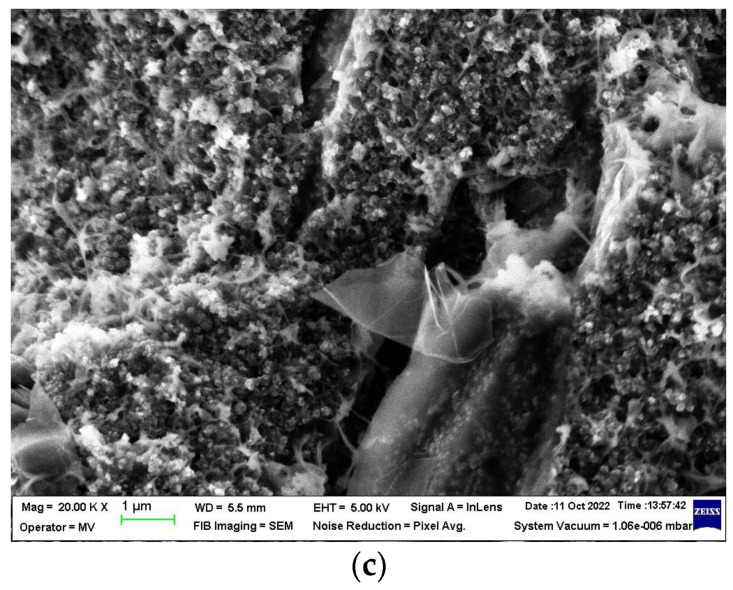
The effect of the preparation procedure on dispersion state of the conductive particles in a polymeric matrix: (**a**) (CB + Gr)/HD composite prepared by melt extrusion; (**b**) (CB + Gr)/HD composite prepared by mold pressing of a powder mixture; (**c**) (CB + Gr)/(LLD + HD) composite prepared by mold pressing of a powder mixture.

The Figure 6 shows the effect of processing on composite morphology: melt blending vs. room temperature physical blending of components in powder state [25]. For (CB + Gr)/HD, the morphologies of these samples appear significantly different (Figure 6a,b): the conductive particles in the case of the sample prepared by physical blending seem to be better segregated at the surface of the polymer phase. Hence, the formation of the conductive paths would be much easier. Indeed, the conductivity of the freshly prepared sample by dry physical mixing of powder components was considerably higher, which suggests a comparison of the thermal effects, namely, the curves (1) and (4) (Figure 5). Therefore, the same mixing procedure was applied for preparation of (CB + Gr)/(LLD + HD) composite (Figure 6c). It can be easily observed that the morphology of this sample is very similar to that of the sample (CB + Gr)/HD prepared by the same procedure (Figure 6b).

**Figure 7:**
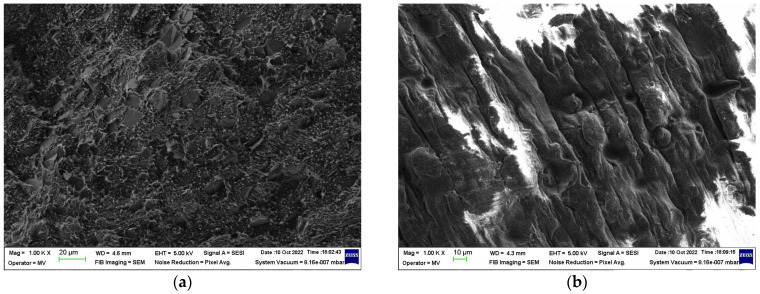

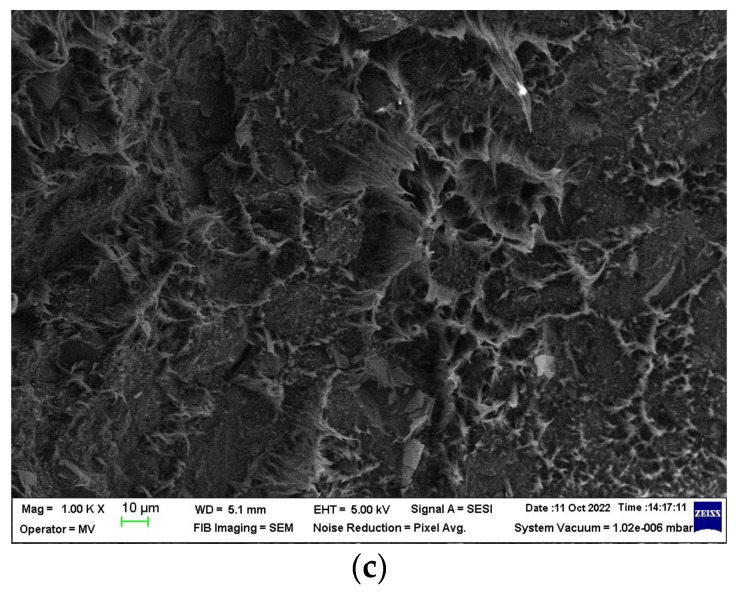
SEM micrographs of (CB + Gr)/HD composite prepared by melt blending followed by: (**a**) press molding; (**b**) injection molding; (**c**) micrography of sample CB + Gr)/(LLD + HD).

Another aspect of processing is comparatively illustrated in Figure 7 in the case of (CB + Gr)/HD composite prepared by melt blending: the sample in Figure 7a was subsequently pressed between two heated plates at 160 °C (press molding), using a spacer of 0.7 mm, while the sample in Figure 7b was prepared by injection molding [29]. While this sample presented conductivity (evolving from poor to high, as described above), the injected sample is practically non-conductive. When re-formed by pressing in the same conditions, the injected sample became conductive at the same level to the sample formed directly by press molding (from pellets of composite). This behavior would be understood if we compare the samples morphologies as they are seen from SEM analysis (Figure 7): the press mold sample appears as a continue material with moderate luminosity, while the injected mold sample appears as a stratified material, possibly due to the fine trepidations related to the injection molding. As a result, the sample present higher luminosity, suggesting that conductive channels may exist within each layer, but they are not extended between two neighboring layers due to the fine interlayer empty spaces. Again, for a same magnification, the aspect (morphology) of the samples (CB + Gr)/HD (Figure 7a) and (CB + Gr)/(LLD + HD) (Figure 7c) are similar due to similar processing procedures (dry mixing in powder state and hot molding).

### 3.2. Resistivity vs. Temperature

#### 3.2.1. Heating Curves

The first observation is that the heating curves ρ-T (resistivity-temperature curves) are sigmoidal, similar to non-isothermal oxidation curves in thermal analysis [26,27], with resistivity instead of the oxidation signal (heat flow, CL), hence similar kinetic parameters can be used to describe the ρ-T curves (Figure 3). The experimental observations are discussed below for each type of composite material, then a comparison of these materials is presented.

**Figure 8:**
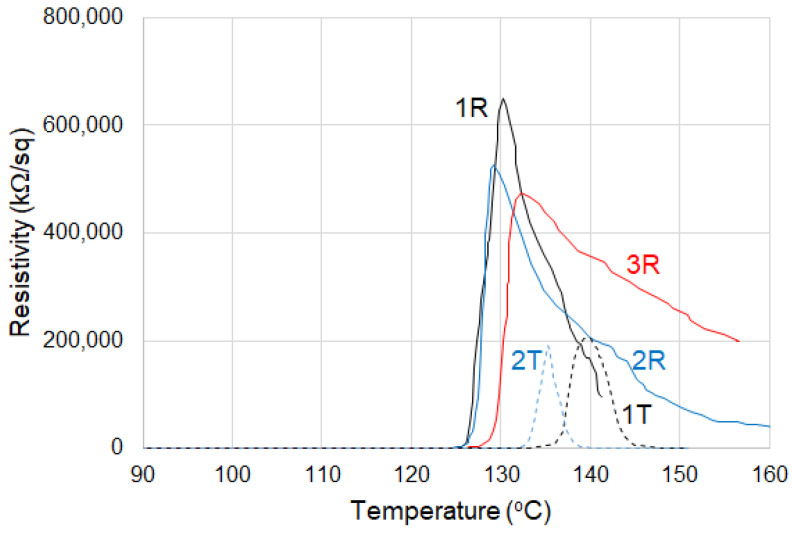
Resistivity vs. temperature for (CB + Gr)/HD samples on repeated temperature cycles, heating portions: 1T, 2T—thermoplastic (D = 0 kGy) at first and second cycle, respectively; 1R, 2R, 3R—crosslinked (D = 150 kGy), cycles 1, 2 and 3, respectively.

For both thermoplastic and crosslinked (CB + Gr)/HD composites, the ρ_max_ values decreased with the number of cycles (Figure 8, Table 2). The onset values for thermoplastic samples decreased with the number of cycles from 139 °C to 133 °C while for the crosslinked material, an inverse trend is observed. Hence, the onset values tend to reach a same value (of ~130 °C) for both thermoplastic and crosslinked materials submitted to repeated thermal cycles. T_max_ data in Table 2 suggest a similar behavior. The slope of resistivity increase appears lower at the first cycle, but it presents higher and comparable values for the further two cycles.

The PTC effect tends to decrease slightly for the crosslinked material from the first to the third cycle due to increased values of ρ_0_ and lower values of ρ_max_. This behavior would be related to the thermo-oxidative degradation of the polymer matrix due to repeated exposure at elevated temperatures. However, for the thermoplastic material, the intensity of PTC effect increased with the number of cycles because the room temperature values of resistivity were lower after the former cycle. Possibly, the thermooxidative degradation is not the single factor affecting the electric properties of the materials subjected to multiple thermal cycles. The oxidation rate of HDPE was reported to be reduced in presence of CB and Gr mixture [22], through several possible mechanisms, namely, (i) direct annihilation of oxidation transient species produced by different active groups on CB surface [30,31], (ii) free radicals trapping by fullerene or fullerene-like structures on CB surface [30,32], and (iii) decrease in oxygen permeability within the amorphous phase induced by carbon particles [4].

In general, the ρ–T curves suggest (Figure 8) an increase in reproducibility with increasing the number of thermal cycles in agreement with previously reported data on different other PTC materials with HDPE matrix [2]. As compared to the other literature data on thermoplastic CB/HD composites, the stability and the reproducibility of the ρ–T curves (see for example [33]) appears higher with our materials (both HD and LLD + HD), possibly due to the benefic influence of the blend of conductive fillers used.

**Figure 9:**
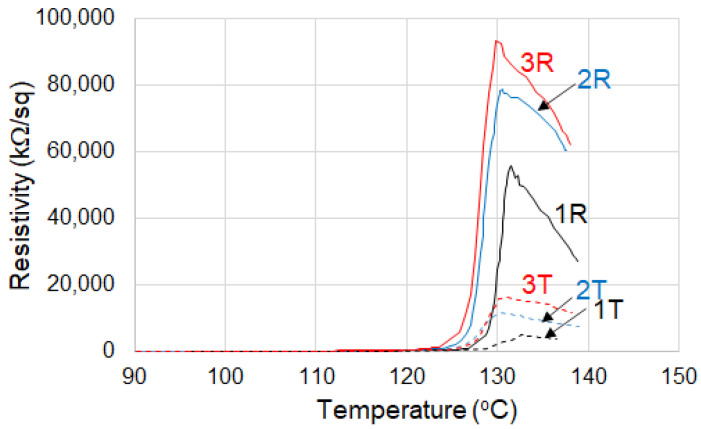
Resistivity vs. temperature for (CB + Gr)/(LLD + HD) samples on repeated temperature cycles, heating portions: 1T, 2T, 3T—thermoplastic (D = 0 kGy) cycles 1, 2, 3, respectively; 1R, 2R, 3R—crosslinked (D = 150 kGy), cycles 1, 2 and 3, respectively.

The ρ–T curves for thermoplastic and crosslinked (CB + Gr)/(LLD + HD) composites are shown in Figure 9, while the parameters describing the kinetics of resistivity increase with temperature are shown in Table 3. The curves at cycles 2 and 3 are closer to each other for the crosslinked material, suggesting more reproducibility, in agreement with previously reported data for radiation-crosslinked materials (see for example [2] for CB/HD and Gr/(LLD + HD) data reported by [17]).

T_max_ and T_onset_ are shifted toward lower temperatures, especially after the first cycle. This effect (attributable to either thermo-oxidative degradation or other structural changes as already mentioned above for (CB + Gr)/HD) would be seen as favorable for device security in limitation/switching applications if we take into account the increase in ρ_max_ with the number of cycles. In any case, these changes are lower as compared to the other literature data, suggesting a more stable network in our case.

For (CB + Gr)/(LLD + HD), the rate of resistivity increase, calculated as the slope of the leading edge of the resistivity peak, is significantly higher for the crosslinked samples as compared to the thermoplastic ones (Table 3).

It is obvious that the resistivity peaks are significantly higher for the crosslinked materials compared to the corresponding thermoplastic ones. The ρ_max_ values also increased with the number of cycles for both thermoplastic and crosslinked materials, but the ρ_max_ values are much higher for the crosslinked samples.

The intensities of the PTC effects are significantly higher (around one order of magnitude) for the crosslinked materials as compared to the thermoplastic ones. However, for the crosslinked materials, the PTC effect tends to slightly decrease as increasing the number of thermal cycles because of increased ρ_0_ values, while an opposite trend is observed for both thermoplastic composites. These opposite behaviors are caused by increase in higher extent of room temperature resistivities with the number of cycles for crosslinked composite as compared to the thermoplastic one (Table 2 and Table 3).

For the crosslinked (CB + Gr)/HD composite, the decrease in the resistivity after exceeding the T_max_ presented two slopes suggesting the occurrence of two processes: one is more rapid and is produced immediately after T_max_, while the second is slower and covers a wider temperature range (Table 2). The slopes of both processes decreased with the number of cycles, and their values became comparable and considerably lower for cycles 2 and 3 as compared to the first cycle. This behavior reflects a decrease in NTC effect after the first thermal cycle. In the case of the thermoplastic material, the resistivity decreased sharply after T_max,_ (Figure 8) until a flat region with low resistivities (~100 kΩ) is reached, suggesting a strong NTC effect. Due to this behavior, the resistivity peak of the thermoplastic material appears more symmetric as compared to the crosslinked one (Figure 8).

The onset temperature appears a little higher for HDPE-matrix samples as compared to the blend ones, especially at the first cycle and for the unirradiated samples, while for the irradiated samples, the onset temperature values are practically similar for HD and (LLD + HD) composites, possibly due to increased similarity of both matrices induced by crosslinking.

The slopes values of ρ increase are significantly higher for HDPE composites than for (LLD + HD) ones, and the ρ_max_ values are considerably higher as well. The intensities of the PTC effects are, therefore, much higher with HDPE composites, especially with the radiation-crosslinked material.

Note that the slope values as calculated by formula (1) correspond to temperature coefficient of resistivity (see for example [34]). Unless the NTC effect, for PTC materials’ TCR has negative values. The slope values represent in our case the maximum of TCR values because the variation of ρ or (ρ/ρ_max_) with temperature is typically not linear for PTC materials.

In addition, the resistivity peak of thermoplastic (CB + Gr)/(HD) appears shifted to lower temperatures to a greater extent than for (CB + Gr)/(LLD + HD) (Figure 8 and Figure 9). For the (CB + Gr)/(LLD + HD) crosslinked samples, the peak becomes wider as the number of cycles increased (Figure 8), while for CB + Gr)/HD, this effect is considerably weaker (the ratio height/width remains practically constant).

The peak intensities (ρ_max_) are lower for (CB + Gr)/(LLD + HD) composite than the (CB + Gr)/(HD) by more than one order of magnitude. For both thermoplastic and crosslinked (CB + Gr)/HD material, the ρ_max_ value tends to decrease by thermal cycling, a behavior which is different to that observed for (CB + Gr)/(LLD + HD) composites and also differing to the above-mentioned literature data ([2] for CB/HD and [15] for CF/HD systems). After three thermal cycles, the ρ_max_ value of crosslinked (CB + Gr)/(LLD + HD) composite became comparable to that of thermoplastic (CB + Gr)/HD.

The intensity of the NTC effects seem to be higher than in the above-mentioned literature cases, where a flat portion of high temperature heating curve is described ([2,15,17]), illustrating the possible role of the composition, conductive phase, type, and blending conditions on the PTC and NTC behavior of conductive composites.

**Figure 10:**
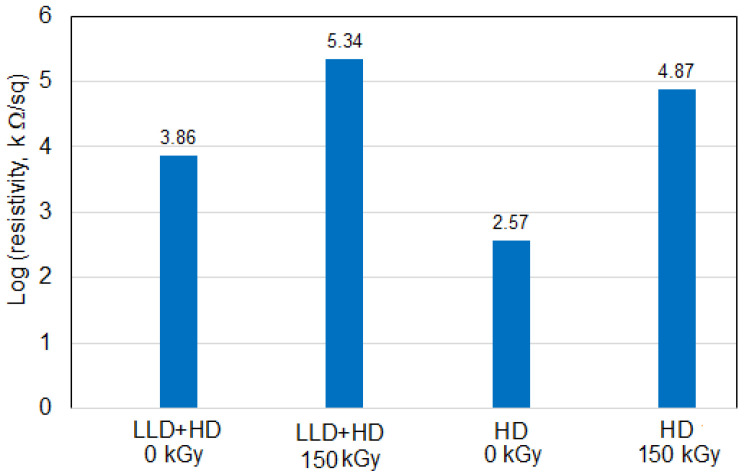
Resistivity at 10 °C from the T_max_ on heating (2nd cycle) of the studied composites.

It can be observed that the resistivity of the thermoplastic (CB + Gr)/HD material decreased strongly as the temperature increased (strong NTC effect), while the crosslinked sample presented only limited decrease in resistivity in molten state (Figure 8). For example, the resistivity at 150 °C (upon heating, 2nd cycle) was ~150 kΩ/sq for the thermoplastic material vs. 74,800 kΩ/sq for the crosslinked sample. This behavior illustrates that crosslinking suppressed significantly the NTC effect for (CB + Gr)/HD material. The wider peaks of resistivity observed for both thermoplastic and crosslinked CB + Gr)/(LLD + HD) and for crosslinked (CB + Gr)/HD, as compared to thermoplastic (CB + Gr)/HD, would be interpreted in a similar manner (see a comparison of resistivity values on heating in Figure 10).

As compared to the similar composite samples with HDPE matrix, the blend LLD + HD induced lower ρ_max_ values, wider resistivity peaks and lower PTC (Table 2 and Table 3, Figure 8 and Figure 9), but lower NTC also, even in thermoplastic state (Figure 10).

#### 3.2.2. Cooling Curves

**Figure 11:**
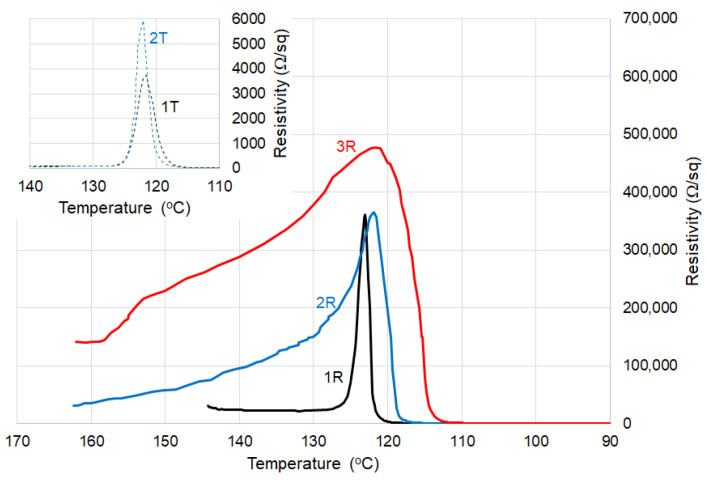
Resistivity vs. temperature for (CB + Gr)/HD samples on repeated temperature cycles, cooling portions: 1T, 2T– thermoplastic (D = 0 kGy) cycles 1 and 2 respectively; 1R, 2R, 3R—crosslinked (D = 150 kGy), cycles 1, 2 and 3, respectively. Note: because of weak intensity of the peaks of thermoplastic material. These curves are shown in the inset.

**Figure 12:**
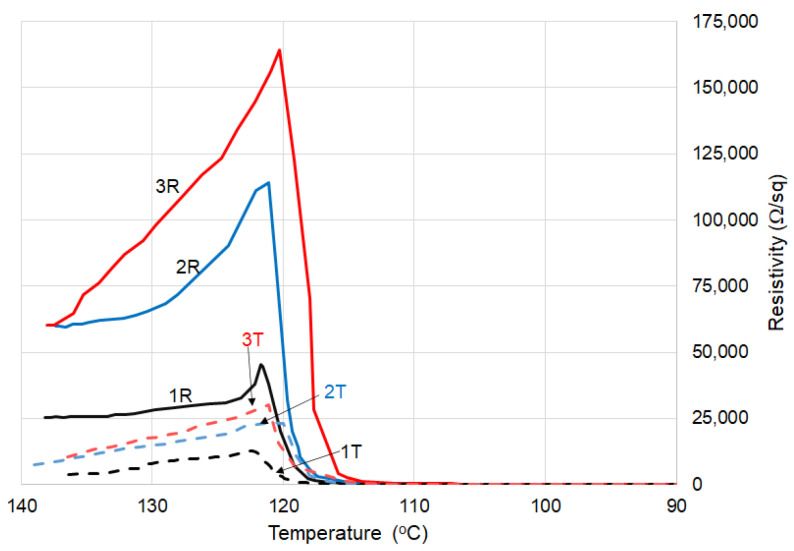
Resistivity vs. temperature for (CB + Gr)/(LLD + HD) samples on repeated temperature cycles, cooling portions: 1T, 2T, 3T—thermoplastic (D = 0 kGy) cycles 1, 2, 3, respectively; 1R, 2R, 3R—crosslinked (D = 150 kGy), cycles 1, 2 and 3, respectively.

The cooling curves are presented in Figure 11 and Figure 12 for (CB + Gr)/HD and (CB + Gr)/(LLD + HD) composites, respectively. The thermoplastic (CB + Gr)/HD presents again a distinct behavior as compared to other materials: the peak is sharp and symmetric while, for crosslinked (CB + Gr)/HD and (CB + Gr)/(LLD + HD), the peaks are clearly asymmetric with slow increase in resistivity in molten state and sharp decrease in solid state.

It can be observed that the resistivity of the HD thermoplastic composites remained low for a relatively long period after heating cease, while other ρs increased with a smoother slope at the beginning followed by a more abrupt region as the temperature approached the T_max_ value. Thus, an onset temperature (T′_onset_) of ρ increase can be defined as the intersection point of the flat (or slightly inclined) region at higher temperatures and the sharply increasing portion of the leading edge (when approaching the peak). The T′_onset_ values of the studied materials are shown in Table 4 and Table 5. While for thermoplastic HD composite, T′_onset_ values are practically unchanged for the first and second cycle, and a significant increase with the number of cycles is observed for crosslinked (CB + Gr)/HD one. Increased values of T′_onset_ signify that a melt material become resistive, on cooling, earlier than a material with lower T′_onset_ values; this behavior would be related with lower NTC and higher PTC properties of such a material. Hence, the increase in T_onset_ values for crosslinked (CB + Gr)/HD suggests an improvement of the electrical properties of this material induced by thermal cycling (assuming that NTC effect is undesired for our case). It can be observed that (CB + Gr)/(LLD + HD) composites present this behavior even in thermoplastic state (Figure 12).

For thermoplastic HD composites, as well as for crosslinked (CB + Gr)/HD at the first cycle, the rate of resistivity increase in molten state can be described by a single slope, while for others, two slopes can be defined for each process, suggesting two mechanisms of decay of conductive paths. This behavior could be related to the existence of two conductive powders with different aspect ratios, CB particles are spherical, while graphite ones are platelike [22], hence they would impart conductivity by different mechanisms [5]. Another factor would be the nature of the polymer matrix: the linear macromolecules allow easier movement of conductive particles and thus allow easier restoration/interruption of conductive paths while, in the case of crosslinked polymers, the mobility of the conductive particles is lower.

It is obvious also that ρ_max_ values are considerably higher for the crosslinked materials, a behavior which is similar to that observed upon cooling. Repeated cycles produced increased and wider resistivity peaks for crosslinked (CB + Gr)/HD and (CB + Gr)/(LLD + HD).

After the ρ_max_ value is reached, the resistivity dropped abruptly until values of hundred kΩ/sq, then the resistivity decreased slowly until few kΩ s were observed at r.t. The slopes of ρ decay (the rate of resistivity decrease) increased with the number of cycles for thermoplastic (CB + Gr)/HD, which remained practically unchanged for thermoplastic (CB + Gr)/(LLD + HD), but tended to decrease for crosslinked composites, especially for the (CB + Gr)/HD one (Table 4 and Table 5). This behavior would be related, as well, to limited mobility of conductive particles in crosslinked polymers. Excepting thermoplastic (CB + Gr)/HD, all other materials presented slightly higher values of r.t. resistivity after each thermal cycle suggesting a certain “ageing” process.

### 3.3. DSC Measurements

**Figure 13:**
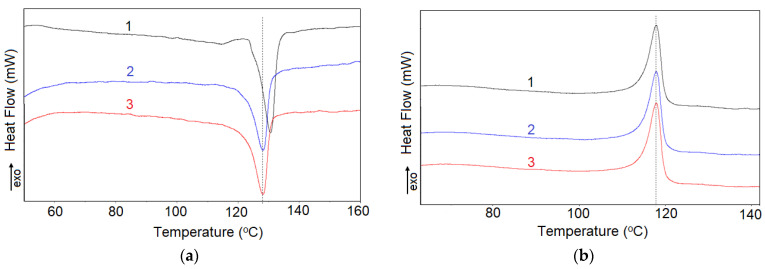
DSC curves from three repeated heating/cooling cycles between r.t. and 170 °C of thermoplastic (CB + HD)/(LLD + HD) composite: (**a**) heating curves (5 °C /min, air); (**b**) cooling curves (5 °C /min, air).

DSC measurements aimed to check if the structural changes induced by repeated cycles in air in DSC furnace would be related to the above-discussed parameters of resistivity vs. temperature curves. The typical recorded heating and the cooling curves are presented in Figure 13. Note that, because the aim of these measurements was to correlate the parameters of the DSC curves to ρ-T curves to melting and crystallization data from DSC, and to detect eventual changes induced by repeated thermal cycles, the samples were measured in their initial state, as resulted from molding, without any treatment for erase their initial thermal history (as usual when the intrinsic melting and crystallization behaviors are assessed, see for example reference [35]).

It was observed that for both thermoplastic and crosslinked (CB + Gr)/(LLD + HD) composites, the heating curves at the first cycle differs from others by presence of a peak at ~114.5 °C (it disappeared to further 2nd and 3rd cycles) and a T_max_ value of ~130.7 °C which subsequently decreased to ~128.1 °C. As the melting peak (for the 2nd and 3rd cycles) is unique, without shoulders or secondary peaks, it can be concluded that the LLD/HD blend is homogeneous. The small peak at ~114.5 °C, on the peak at first cycle, was related to a pseudo-crystalline phase possibly resulted on composite molding [22]. It seems to be related to the presence of HDPE. In the case of thermoplastic (CB + Gr)/HD composite, the shoulder on the main peak persists to further cycles while, for the crosslinked material, this shoulder is visible in the cooling curve at first cycle only, and not in the further ones. A diminution in crystallinity (calculated from DSC) of ~10% is also produced after the first cycle for crosslinked (CB + Gr)/HD as compared to less than 2% for (CB + Gr)/(LLD + HD) composites (either crosslinked or thermoplastic). For thermoplastic (CB + Gr)/HD composite, the drop in crystallinity after the first cycle is ~15%, but the decrease continued to further cycles, suggesting that thermoplastic HDPE network would be less stable than (CB + Gr)/(LLD + HD) one.

In general, the parameters of the heating curves of (CB + Gr)/(LLD + HD) shown in Table 6 were practically the same for the 2nd and the 3rd cycles, and differed slightly from those of the first cycle. In the case of (CB + Gr)/HD composites, the previous statements are especially valid for the crosslinked material, while the thermoplastic one appears less stable at repeated cycles test (Table 7). However, as the melting temperature does not practically change with repeated DSC cycles, the observed changes cannot be attributed to oxidative (chemical) degradation, but rather to molecular rearrangements which affect the crystallinity content.

**Figure 14:**
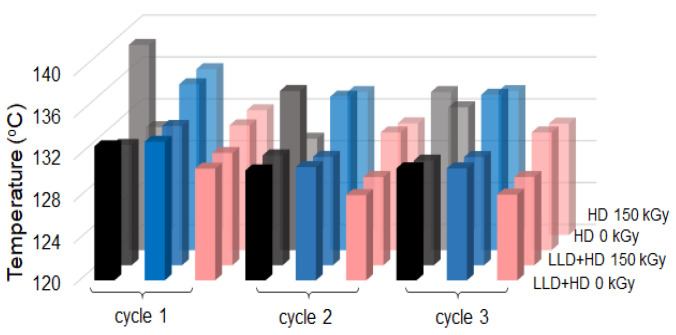
Correlation of T_max_ (■) from heating ρ-T curves with T_offset_ (■) and T_max_ (■) from heating DSC curves. The color intensities decrease, from front to back, in order to facilitate the comparison of the parameters.

The behavior of the (CB + Gr)/(LLD + HD) samples at the first heating cycle (Table 6) are similar to those of the resistivity variations with the temperature (see T_onset_, T_max_, T_offset_ data in Table 3) in the sense that the parameters of the first cycle are different from those of other two cycles which are practically equal. The resistivity peak (T_max_) values are close to the T_offset_ from DSC ones (Figure 14), suggesting that the maximum of the resistivity is reached when the crystallinity completely disappear.

In the case of thermoplastic HDPE composite, the temperature of reaching ρ_max_ is closer to T_offset (DSC)_, that is the maximum of resistivity corresponds to complete molten state of the matrix (Figure 14). This behavior resembles to (CB + Gr)/(LLD + HD) composites. For the crosslinked (CB + Gr)/HD composite, the temperatures of ρ_max_ are better correlated to T_max (DSC)_, meaning that there is still crystallinity within the system when the ρ_max_ value is attained. In general, the resistivity data seem to be poorly correlated with DSC ones for (CB + Gr)/HD composites as compared to (CB + Gr)/(LLD + HD) ones.

The cooling curves are practically the same for all three cycles both in the case of thermoplastic and crosslinked (CB + Gr)/(LLD + HD) composites (Figure 13b, Table 7). In the case of crosslinked composite, the crystallization occurs slightly earlier, as the higher values of T_max_ and T_onset_ suggest, as compared to the thermoplastic material, due to lower mobility of crosslinked polymer chains. However, the duration of crystallization process remains practically the same, as suggest the values of difference between the average T_onset_ and T_offset_ values (${∆}_{c}={\;\bar{T}}_{\mathrm{onset}}-{\;\bar{T}}_{\mathrm{offset}}),$ which are equal to 5.67 °C for thermoplastic and 5.72 °C for crosslinked composite. HDPE composites behave in general similarly (Table 8 and Table 9), with the only difference of the above-mentioned shoulder. The Δ_c_ parameter has similar values to (CB + Gr)/(LLD + HD), namely, 5.67 °C for thermoplastic and 5.74 °C for crosslinked (CB + Gr)/HD composites, suggesting that crystallization processes are similar for both materials.

**Figure 15:**
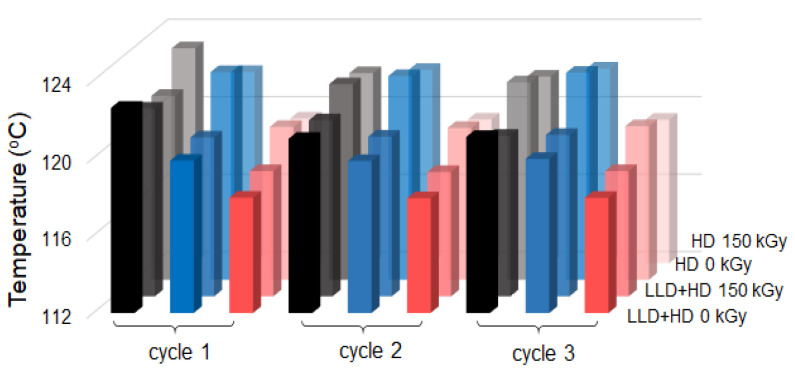
Correlation of T′_max_ (■) from cooling ρ-T curves with T_c_ (■) and T_offset_ (■) from cooling DSC curves. The color intensities decrease from front to back, in order to facilitate the comparison of the parameters.

Concerning the correlation between the parameters of ρ-T and DSC cooling curves, for (CB + Gr)/(LLD + HD) composites, T′_max_ values (considered as relevant for PTC properties) are closer to T_onset_ ones from DSC (Figure 15). This result is consistent with that from heating curves (Figure 14) in the sense that the maximum of the resistivity corresponds to the start of crystallization process (the system does not contain crystallinity, but it is going to have it immediately). For (CB + Gr)/HD composites, T′_max_ values are also close to the T_onset_ (DSC), meaning that the melt HDPE matrix behave similarly to (LLD + HD) one. Note also that the values of peak temperatures (T_max_) from DSC curves did not indicate the occurrence of some structural changes during the multiple thermal cycles, hence no chemical degradation can be supposed neither heating nor cooling.

The correlations of the behavior of the studied composites in DSC and ρ-T measurements would be related to major changes in conductive particles distribution within the liquid polymer matrix. However, it is not clear why the considerably rise of resistivity peak height in the case of crosslinked composites is observed. If radiation used for crosslinking bonds strongly, the CB particles on polymer chains, matrix dilatation in molten state should enable stronger dilatation effects. Thermally induced supplementary fixation of CB particles during the repeated cycles would result in higher resistivity values for (CB + Gr)/(LLD + HD), both in crosslinked and in thermoplastic state, as well as for crosslinked (CB + Gr)/HD composite. For Gr particles (where no significant interactions with the polymer chains are expectable), the rearrangement in the molten state is more probable mechanism. Hence, the higher values of the crystallinity and T_max_ at the first cycle can be assigned to molecular rearrangements during the sample molding and subsequent storage. Their evolution is in the same direction with the thermal parameters of the resistivity, but not with the resistivity values themselves.

### 3.4. Temperature Self-Regulation Behavior

**Figure 16:**
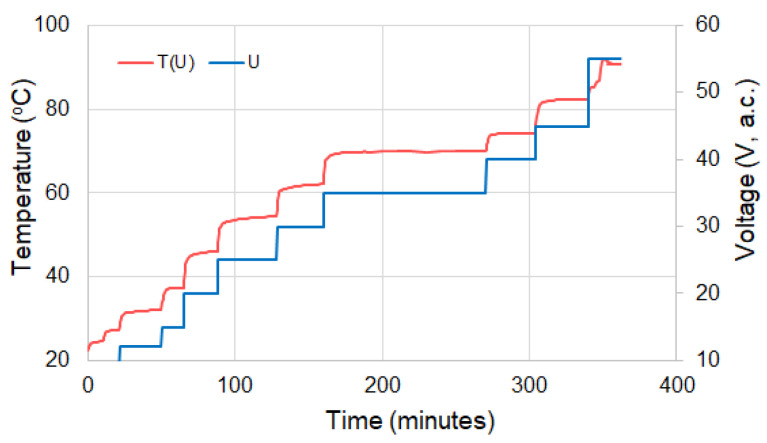
T_s_ vs. time and applied voltage for a (CB + Gr)/HD composite.

**Figure 17:**
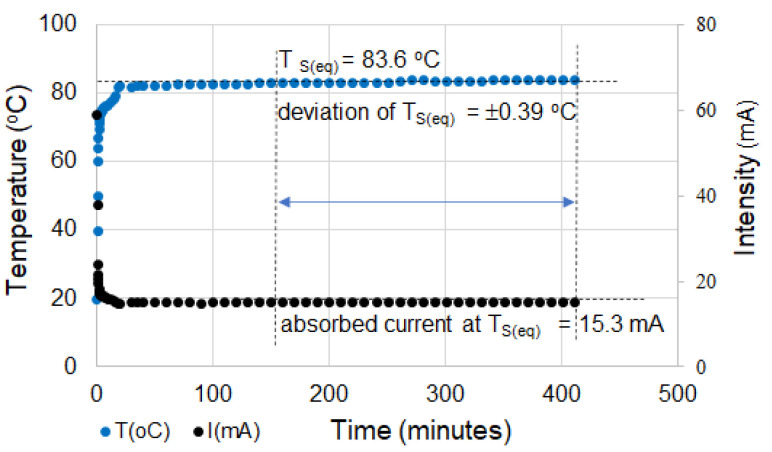
T_s_ vs. time and current intensity through the sample vs. time for a (CB + Gr)/HD composite (applied voltage, 55 V).

**Figure 18:**
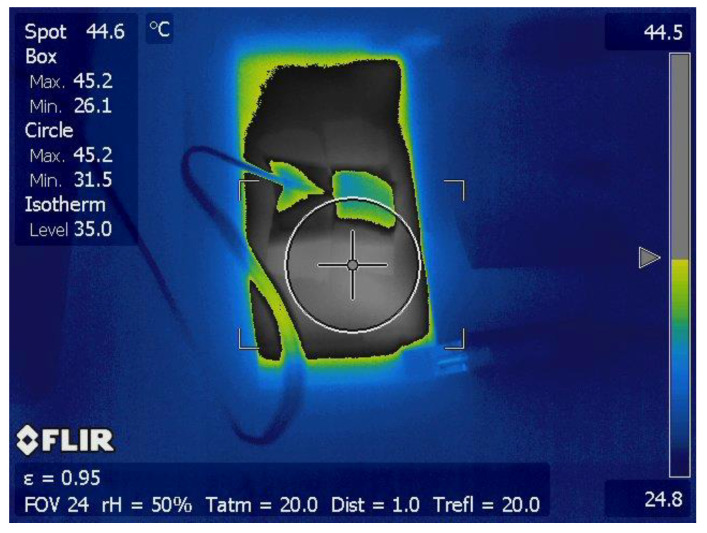
Thermal imaging image of a plate made of thermoplastic composite during heating (Ts not reached). The coil on the surface of the plate is a cable of a thermocouple attached to the sample.

The operation of the studied composites as self-regulating heating elements is illustrated in the Figure 5, Figure 16, Figure 17 and Figure 18 and is based on the PTC effect shown in Figure 8 and Figure 9. Practically, the jump of 4–5 orders of magnitude of the resistivity, from a few kΩ/sq to values of the order of 10^4^–10^5^ kΩ, enables a clear transition of the material from the state of semiconductor to that of electrical insulator, ensuring so the functionality of the element.

The curves of surface temperature (T_s_) vs. time (Figure 5) show, as already mentioned, the effect of some treatments on T_s_. It can be noted that although the surface equilibrium (plateau) temperature (T_s(eq)_) is considerably lower than T_max_ (from the ρ-T or DSC curves), and the materials show an obvious self-limiting effect of T_s_.

Figure 16 shows that, apart from compositional effects and those regarding treatments applied during or after processing, the T_s_ value can also be controlled with the help of the voltage (electric field) applied between the electrodes of the element. This feature allows these materials to be used over a much wider temperature range, not just near T_max_. In this range, where T_s_ < T_max_, the material operates in a regime apparently similar to constant power devices. However, from Figure 17, it can be seen that the self-limiting properties are clearly manifested for very long, practically infinite periods, in which both T_s_ and the intensity of the current passing through the element remain constant.

Figure 18 shows the thermal image of the surface of a heating element in the form of a plate with dimensions of 120 × 100 × 0.8 mm made from (CB + Gr)/HD thermoplastic material (with the electrodes fixed on the 120 mm sides). The uniformity of the temperature distribution on the surface of the element is noticeable, even during heating, when the non-uniformity of the temperature field is expected to be higher, especially for samples with large distances between the electrodes.

## 4. Conclusions

A novel conductive composite material with homogeneous binary polymer matrix of HDPE and LLDPE and mixed conductive fillers (carbon black and graphite) was tested against a composite with similar conductive filler but with HDPE matrix. Even the concentration of the conductive filler was deliberately lower for (CB + Gr)/(LLD + HD), and the properties of this composite are comparable or better to those of (CB + Gr)/HD.

The kinetic parameters of the ρ–T curves (most relevant being T_max_ on heating and T′max on cooling) correlate well with T_offset_ on heating or T_onset_ on cooling from the DSC curves, indicating that the resistivity peak is obtained when the polymer matrix is fully melted. However, for (CB + Gr)/HD, upon heating, the maximum of resistivity corresponds to T_max_ from DSC, i.e., when a certain degree of crystallinity still persists in the system.

When subjected to repeated thermal cycles, the composite (CB + Gr)/(LLD + HD) presented a better electrical behavior than CB + Gr)/HD, with an increase in ρ_max_ values with the number of cycles, as well as less intense NTC effects, both for the crosslinked and thermoplastic samples.

Radiation crosslinking led to increased ρ_max_ values, as well as inhibition of NTC effects in both cases, thus having a clear beneficial effect.

Limitation effects of surface temperature and current intensity through the sample were observed at different voltages, enabling the use of these materials as self-regulating heating elements at temperatures below the melting temperature.

The procedure based on physical mixing of components appears more efficient in imparting lower resistivity in solid state and high PTC effects to the composites, possibly due to the concentration of the conductive particles at the surface of the polymer domains. This heterogeneous distribution of the filler would facilitate the formation of the conductive paths, because a greater number of conductive particles are available on the surface of the polymer domains (the conductive filler would escape easier to be incorporated into the insulating polymer layer). Further work is still necessary to optimize both the procedure of composite synthesis and the properties of such materials.

## Figures and Tables

**Table 1:** List of abbreviations.

Abbreviation	Full Name/Description
PTC	Positive Temperature Coefficient
NTC	Negative Temperature Coefficient
HDPE (HD)	High Density Polyethylene
LLDPE (LLD)	Linear Low-Density Polyethylene
FEF	Fast Extruder Furnace
SEM	Scanning Electron Microscopy
CB	Carbon Black
Gr	Graphite
ρ_V_	Volume resistivity
ρs	Surface resistivity
T_s_	Sample surface temperature
ρ-T	Resistivity-Temperature
X_c_	Crystallinity degree
OOT	Onset Oxidation Temperature
ΔH_m_/ΔH_c_	Melting/Crystallization enthalpies
T_m_/T_c_	Melting/Crystallization temperatures

**Table 2:** Kinetic data of resistivity increase upon the heating of (CB + Gr)/HD composites.

Dose (kGy)	Cycle Number	ρ_0_ (kΩ/sq)	Onset Temperature (°C)	Slope of ρ Increase (K^−1^)	T_max_ (°C)	ρ_max_·10^−5^ (kΩ/sq)	PTC Intensity lg(ρ_max_/ρ_0_)	Slope of ρ Decrease (K^−1^)
0	1	3.50	137.0	0.54	139.6	2.32	4.83	−0.26
	2	1.49	133.2	0.49	135.2	1.92	5.11	−0.47
	3	1.23	133.1	0.47	135.1	1.90	5.19	−0.47
150	1	1.11	126.1	0.27	130.3	6.51	5.77	−0.14 −0.07
	2	2.73	127.1	0.64	129.2	5.26	5.28	−0.09 −0.03
	3	3.77	129.4	0.51	132.2	5.10	5.10	−0.04 −0.03

**Table 3:** Kinetic data of resistivity increase upon the heating of (CB + Gr)/(LLD + HD) composites.

Dose (kGy)	Cycle Number	ρ_0_ (kΩ/sq)	Onset Temperature (°C)	Slope of ρ Increase (K^−1^)	T_max_ (°C)	ρ_max_·10^−4^ (kΩ/sq)	PTC Intensity lg(ρ_max_/ρ_0_)	Slope of ρ Decrease (K^−1^)
0	1	4.12	122.2	0.25	132.8	0.481	3.07	−0.05
	2	6.85	126.3	0.26	130.5	1.143	3.22	−0.05
	3	8.53	128.1	0.35	130.7	1.610	3.28	−0.04
150	1	4.49	129.0	0.32	131.5	5.560	4.09	−0.04
	2	11.2	126.5	0.37	130.5	7.860	3.85	−0.03
	3	14.47	126.7	0.38	130.0	9.320	3.81	−0.04

**Table 4:** Kinetic data of resistivity change on cooling of (CB + Gr)/HD composites.

Dose (kGy)	Cycle Number	T′_onset_ (°C)	Slope of ρ Increase in Molten State (K^−1^)	ρ′_max_⋅10^−4^ (kΩ/sq)	T′_max_ (°C)	Slope of ρ Decay in Solid State (K^−1^)	T_offset_ (°C)	ρ_r.t._ at the Cycle End (kΩ/sq)
0	1	124.6	−0.41	0.375	121.5	0.28	118.7	1.54
	2	124.2	−1.07	0.594	122.1	0.50	120.2	1.23
	3	124.1	−1.10	0.602	122.2	0.55	120.3	1.19
150	1	125.0	−0.53	36.06	123.1	1.09	122.0	2.79
	2	127.2	−0.10 −0.01	36.50	121.81	0.33	119.0	3.83
	3	134.8	−0.03 −0.01	47.65	121.64	0.20	114.4	8.7

**Table 5:** Kinetic data of resistivity change upon the cooling of (CB + Gr)/(LLD + HD) composites.

Dose (kGy)	Cycle Number	T′_onset_ (°C)	Slope of ρ Increase in Molten State (K^−1^)	ρ′_max_⋅10^−4^ (kΩ/sq)	T′_max_ (°C)	Slope of ρ Decay in Solid State (K^−1^)	T′_offset_ (°C)	ρ_r.t._ at the End of Cycle (kΩ/sq)
0	1	134.1	−0.06	1.255	122.6	0.43	119.8	6.85
	2	>139	−0.04	2.340	121.0	0.47	118.9	8.94
	3	>137	−0.04	3.030	121.1	0.41	118.9	13.15
150	1	133.7	−0.04 −0.18	4.55	121.7	0.51	119.2	11.22
	2	129.2	−0.02 −0.05	11.40	121.1	0.53	119.2	16.02
	3	135.9	−0.03 −0.08	16.43	120.3	0.30	117.1	18.81

**Table 6:** Kinetic parameters of the melting curve of (CB + Gr)/(LLD + HD).

Dose (kGy)	Cycle	T_m_ (°C)	ΔH_m_ (J/g)	T_onset_ (°C)	T_offset_ (°C)
0	1	130.66	122.5	124.6	133.24
	2	128.14	117.5	121.4	130.82
	3	128.18	117.4	121.3	130.70
150	1	130.72	122.2	123.7	133.30
	2	128.43	119.9	121.6	130.35
	3	128.44	119.5	121.6	130.33

**Table 7:** Kinetic parameters of the crystallization curve of (CB + Gr)/(LLD + HD).

Dose (kGy)	Cycle	T_c_ (°C)	ΔH_c_ (J/g)	T_onset_ (°C)	T_offset_ (°C)
0	1	117.96	−106.6	119.87	114.28
	2	117.93	−106.6	119.85	114.17
	3	117.95	−106.7	119.96	114.22
150	1	118.47	−106.4	120.21	114.56
	2	118.43	−106.2	120.25	114.45
	3	118.47	−106.2	120.34	114.57

**Table 8:** Kinetic parameters of the melting curve of (CB + Gr)/HD.

Dose (kGy)	Cycle	T_m_ (°C)	ΔH_m_ (J/g)	T_onset_ (°C)	T_offset_ (°C)
0	1	131.92	183.4	124.56	135.84
	2	131.24	156.2	122.84	134.66
	3	131.24	149.4	122.68	134.85
150	1	131.92	175.5	124.56	135.84
	2	130.67	157.1	121.53	133.65
	3	130.64	157.2	121.57	133.75

**Table 9:** Kinetic parameters of the crystallization curve of (CB + Gr)/HD.

Dose (kGy)	Cycle	T_c_ (°C)	ΔH_c_ (J/g)	T_onset_ (°C)	T_offset_ (°C)
0	1	119.90	−128.7	122.73	112.31
	2	119.84	−122.4	122.52	112.59
	3	119.93	−122.2	122.69	112.28
150	1	119.47	−128.8	121.88	112.12
	2	119.42	−121.4	121.98	112.32
	3	119.41	−121.0	122.05	112.27

## Data Availability

The data presented in this study are available on request from the corresponding author.
